# Commentary on ‘Totally laparoscopic versus laparoscopy-assisted distal gastrectomy: the KLASS-07, a randomized controlled trial’

**DOI:** 10.1097/JS9.0000000000001687

**Published:** 2024-05-20

**Authors:** Banwari L. Bairwa, Ayush Anand, Nathnael A. Woldehana, Prakasini Satapathy, Shilpa Gaidhane, Quazi S. Zahiruddin, Sarvesh Rustagi

**Affiliations:** a MP Birla Hospital and Research Center, Chittorgarh; b MediSurg Research, Darbhanga; c Center for Global Health Research, Saveetha Medical College and Hospital, Saveetha Institute of Medical and Technical Sciences, Saveetha University, Chennai; d One Health Centre (COHERD), Jawaharlal Nehru Medical College; e South Asia Infant Feeding Research Network (SAIFRN), Division of Evidence Synthesis, Global Consortium of Public Health and Research, Datta Meghe Institute of Higher Education, Wardha; f School of Applied and Life Sciences, Uttaranchal University, Dehradun, Uttarakhand, India; g Medical Laboratories Techniques Department, Al-Mustaqbal University, Hillah, Babil, Iraq; h B.P. Koirala Institute of Health Sciences, Dharan, Nepal; i Myungsung Medical College, Addis Ababa, Ethiopia; j Johns Hopkins Bloomberg School of Public Health, Baltimore, Maryland, USA

*Dear Editor,*

We would like to congratulate Park *et al*.^1^ on the publication of the manuscript titled ‘Totally laparoscopic versus laparoscopy-assisted distal gastrectomy: the KLASS-07, a randomized controlled trial.’ The study is robust in its design and provides valuable insights into the comparison between totally laparoscopic distal gastrectomy (TLDG) and laparoscopy-assisted distal gastrectomy (LADG). However, several limitations need to be addressed to understand the broader implications of this research. First, the study exclusively focused on patients with clinical stage I gastric cancer. This restricts the generalizability of the findings to more advanced stages of gastric cancer, where the benefits of TLDG over LADG might differ due to the increased complexity of the surgery and potentially higher morbidity. Second, all participating surgeons were highly experienced in both TLDG and LADG. This level of expertise may not reflect the average skill set found in the broader surgical community, particularly in regions where laparoscopic techniques are not as prevalent or developed. Third, although the study presents data on the quality of life up to 1-year post-operation, longer follow-up would be beneficial to assess if the noted benefits in quality of life (QoL) extend beyond this period, especially considering the life expectancy of patients after successful cancer surgery. Fourth, the exclusion of Billroth I (BI) reconstruction may influence the applicability of results, especially in settings where BI is commonly practiced due to anatomical or preference reasons. Fourth, the study did not provide detailed insights into the learning curve associated with TLDG, which can be steeper compared to LADG. Understanding this learning curve is crucial for training and for centers looking to adopt TLDG.

The study findings have profound implications in surgical practice. First, the reduced rates of ileus and pulmonary complications with TLDG suggest that this approach might enhance recovery protocols and shorten hospital stays, a significant advantage in improving healthcare efficiency and patient experience. Second, given the technical demands of TLDG, there is a clear implication that surgical training programs should emphasize advanced laparoscopic skills. Simulation-based training and stepwise learning could be crucial for less experienced surgeons. Third, the transient improvements in QoL after TLDG, particularly in terms of pain and body image, support a more patient-centered approach in surgical decision-making, where patient preferences and perceptions are given considerable weight. Fourth, the findings suggest that while TLDG has specific short-term benefits, the choice between TLDG and LADG should be based on a comprehensive assessment of patient-specific factors, including comorbidities, body habitus, and even patient preferences regarding scar placement and recovery.

These findings should prompt the integration of TLDG into surgical practice. First, these results should be incorporated into national and international guidelines for the surgical treatment of early-stage gastric cancer, emphasizing the role of TLDG for suitable patients^2^. Second, integration into practice should involve a multidisciplinary team, including oncologists, radiologists, and gastroenterologists, to ensure that the surgical approach is part of a holistic treatment plan. Third, surgical departments should consider tracking specific postoperative outcomes like ileus and pulmonary complications as part of their quality improvement programs, particularly when assessing the adoption of TLDG.

Future research should aim to refine patient selection criteria for TLDG, potentially developing predictive models that incorporate patient, tumor, and surgical factors to optimize outcomes, particularly in advanced stages of gastric cancer. The aim should be to provide data on longer-term survival and quality of life, particularly to see if the early benefits in terms of QoL persist and translate into long-term advantages^3^. Also, cost-effectiveness studies comparing TLDG and LADG would be valuable, considering the entire spectrum of costs, including those associated with complications, readmissions, and long-term care needs. Finally, a detailed analysis of the learning curve for TLDG would help in understanding the training needs and the point at which proficiency is achieved compared to LADG.

In conclusion, the KLASS-07 trial is a significant addition to our understanding of laparoscopic techniques for gastric cancer. While the findings advocate for the benefits of TLDG in specific contexts, a balanced approach considering the patient’s overall treatment plan, surgeon’s experience, and institutional capabilities is essential for integrating these insights into routine clinical practice. The future of gastric cancer surgery lies in personalized, precise, and minimally invasive techniques, with TLDG being an important option in the surgical armamentarium.

## Ethical approval

Not applicable.

## Consent

Not applicable.

## Sources of funding

Not applicable.

## Author contribution

B.L.B.: conceptualization, project administration, writing – original draft, and writing – review and editing; A.A.: project administration, writing – original draft, and writing – review and editing; N.A.W., P.S., S.G., Q.S.Z., and S. R.: supervision, validation, and writing – review and editing.

## Conflicts of interest disclosure

The author declares no conflict of interest.

## Research registration unique identifying number (UIN)

Not applicable.

## Guarantor

Banwari Lal Bairwa.

## Data availability statement

Not applicable.

## Provenance and peer review

Not commissioned, externally peer-reviewed.
